# SAP loss limits anti-insulin atypical B cell activation and pro-inflammatory CD8 T cells despite preserved Tfh responses to protect against type 1 diabetes

**DOI:** 10.64898/2026.07.29.741363

**Published:** 2026-08-02

**Authors:** Landon M. Clark, Dudley H. McNitt, Jack C. McAninch, Lindsay E. Bass, Marguerite L. Padgett, Andres F. Moreno, Christina T. Brannon, Casey M. Nichols, Matthew T. Stier, Rachel H. Bonami

## Abstract

SLAM-associated protein (SAP) is required for T follicular helper (Tfh)-B cell interactions that underlie germinal center formation, but it is unclear if SAP governs islet-reactive CD4+ T cell-B cell interactions and downstream pro-inflammatory CD8+ T cell destruction of islets in type 1 diabetes (T1D). To address this question, we utilized the VH125 ^SD^ .NOD mouse model, whereby 1-3% of all B cells bind insulin. Germline *SAP* loss in this model led to reduced T1D incidence and impaired germinal center B cell formation, yet did not alter T follicular helper cell formation or phenotype. *SAP* loss reduced pro-inflammatory and activated insulin-autoreactive B-T interactions and limited anti-insulin B cell proliferation, activation, and upregulation of co-stimulatory molecules otherwise enhanced in the pancreas. Anti-insulin extrafollicular antibody and memory responses following immunization were preserved in VH125 ^SD^ . *SAP ^-/-^* .NOD mice, but activated atypical anti-insulin B cell responses were reduced. Ultimately, *SAP* loss led to reduced pro-inflammatory CD8+ T cell formation and islet-reactive progenitor exhausted CD8+ T cells in pancreata. These data highlight the essential role of SAP in mediating proinflammatory, anti-insulin B-T interactions to support T1D.

## Full Text Availability

The license terms selected by the author(s) for this preprint version do not permit archiving in PMC. The full text is available from the preprint server.

